# Comparable safety and non-inferior immunogenicity of the SARS-CoV-2 mRNA vaccine candidate PTX-COVID19-B and BNT162b2 in a phase 2 randomized, observer-blinded study

**DOI:** 10.1038/s41598-024-55320-1

**Published:** 2024-03-04

**Authors:** Lawrence Reiter, Johann Greffrath, Bian Zidel, Mario Ostrowski, Jennifer Gommerman, Shabir A. Madhi, Richard Tran, Natalia Martin-Orozco, Rajesh Krishnan Gopalakrishna Panicker, Curtis Cooper, Aleksandra Pastrak

**Affiliations:** 1 Providence Therapeutics Holdings Inc., 120-8832 Blackfoot Trail SE, Calgary, AB T2J 3J1 Canada; 2 South African Medical Research Council Vaccines and Infectious Diseases Analytics Research Unit, Faculty of Health Sciences, University of the Witwatersrand, Johannesburg, South Africa; 3 Malton Medical Center, 6870 Goreway Dr., Mississauga, ON L4V 1P1 Canada; 4 Department of Medicine, Immunology, University of Toronto, Medical Sciences Building, Rm 6271. 1 King’s College Circle, Toronto, ON M5S 1A8 Canada; 5 Department of Immunology, Temerty Faculty of Medicine, 1 King’s College Circle, Rm. 7233, Toronto, ON M5S 1A8 Canada; 6 The Ottawa Hospital Viral Hepatitis Program, Division of Infectious Diseases, Department of Medicine, The Ottawa Hospital, University of Ottawa, 75 Laurier Ave. East, Ottawa, ON K1N 6N5 Canada

**Keywords:** Drug discovery, Immunology, Diseases

## Abstract

In the aftermath of the COVID-19 pandemic, the evolution of the SARS-CoV-2 into a seasonal pathogen along with the emergence of new variants, underscores the need for dynamic and adaptable responses, emphasizing the importance of sustained vaccination strategies. This observer-blind, double-dummy, randomized immunobridging phase 2 study (NCT05175742) aimed to compare the immunogenicity induced by two doses of 40 μg PTX-COVID19-B vaccine candidate administered 28 days apart, with the response induced by two doses of 30 µg Pfizer-BioNTech COVID-19 vaccine (BNT162b2), administered 21 days apart, in Nucleocapsid-protein seronegative adults 18–64 years of age. Both vaccines were administrated via intramuscular injection in the deltoid muscle. Two weeks after the second dose, the neutralizing antibody (NAb) geometric mean titer ratio and seroconversion rate met the non-inferiority criteria, successfully achieving the primary immunogenicity endpoints of the study. PTX-COVID19-B demonstrated similar safety and tolerability profile to BNT162b2 vaccine. The lowest NAb response was observed in subjects with low-to-undetectable NAb at baseline or no reported breakthrough infection. Conversely, participants who experienced breakthrough infections during the study exhibited higher NAb titers. This study also shows induction of cell-mediated immune (CMI) responses by PTX-COVID19-B. In conclusion, the vaccine candidate PTX-COVID19-B demonstrated favourable safety profile along with immunogenicity similar to the active comparator BNT162b2 vaccine.

## Introduction

Since emergence of the Severe Acute Respiratory Syndrome Coronavirus 2 (SARS-CoV-2) in 2019, the global count of reported cases of coronavirus disease 2019 (COVID-19) has surpassed 770 million with recorded deaths exceeding 6.9 million worldwide. However, these tolls could be underestimated considering serological and epidemiological studies reporting > 90% seropositivity in populations with diverse vaccine coverages^1^. In response to this challenge, an unprecedented array of vaccine candidates based on both traditional and novel platforms has been developed. Among that multitude of promising vaccine candidates, the emergence of mRNA-based vaccines has redefined the landscape of vaccine development and deployment. The mRNA vaccines have demonstrated remarkable efficacy against severe COVID-19 infection, offering a powerful tool in the fight against the pandemic. Clinical trials evaluating the immunogenicity and safety of mRNA vaccines have consistently reported robust immune responses, including the induction of humoral and cellular immunity^2,3^. The platform's ability to be rapidly reprogrammed to accommodate emerging viral variants is a potent tool against the ever-evolving nature of SARS-CoV-2. The Spike (S) glycoprotein of SARS-COV-2 is the target antigen (Ag) in almost all of these vaccines. It contains a receptor binding domain (RBD) that binds strongly to human angiotensin-converting enzyme 2 (ACE2) receptors, playing a major role in viral attachment, fusion and entry into host cells^4,5^. Neutralizing antibodies (NAb) directed against the S protein were known to provide protection from other highly pathogenic coronaviruses (e.g.: SARS-1, Middle East Respiratory Syndrome) and a similar protective effect was rapidly demonstrated with anti-S antibodies against SARS-CoV-2 infection. Levels of S-binding and neutralizing antibodies are associated with protection against diseases caused by both the ancestral strain and the wide array of variants of concern (VOC) that subsequently emerged^6–8^. NAb immunobridging (i.e. predicting the efficacy of a vaccine candidate based on the NAb response by comparing an investigational vaccine to a similar existing vaccine) has therefore been adopted as an approach to support vaccine approval. In July 2021, vaccine candidate MVC-COV1901 (Medigen Vaccine Biologics Corporation, Taiwan) was one of the first vaccines approved based on immunobridging studies^9^. Consensus position has since been taken up by the Access Consortium, which consisted of regulatory authorities from the UK, Australia, Canada, Singapore, and Switzerland, to accept immunobridging studies as sufficient for authorizing COVID-19 vaccines^10^. Immunobridging has also been used to infer vaccine effectiveness of different age groups and COVID-19 booster shots after demonstration of vaccine effectiveness in a clinical endpoint efficacy trial^11^.

While the COVID-19 pandemic highlighted the importance of robust vaccine development programs, the end of the pandemic and the evolution toward an endemic virus situation, marked by the periodic emergence of new immune escape variants combined with weaning immunity stressed the need to improve, diversify and secure vaccine supplies, especially for older adults and persons who may require annual boosters^12^. Providence Therapeutics Holdings, Inc. (Providence Therapeutics) developed an mRNA vaccine, PTX-COVID19-B, composed of a lipid nanoparticle containing a modified mRNA that encodes for the full-length S protein with glycine in position 614 (G614). Previous studies suggested that full-length Spike with D614G can stabilize the Spike in a pre-fusion conformation that is necessary to induce high titers of nAbs^13–15^. The D614G variant of SARS-CoV-2 was the predominant strain worldwide and this substitution prevailed in current, more transmissible VOC. PTX-COVID19-B vaccine candidate was demonstrated to be safe, well tolerated and immunogenic in a Phase 1 clinical study testing a prime-boost regimen at three different doses in healthy adults^16^. The two doses of 40 µg, 28 days apart vaccine regimen presented the right balance between the immune response and reactogenicity profile and was therefore selected for evaluation in this phase 2 clinical study. This immunobridging study aimed to compare the safety and immunogenicity of PTX-COVID19-B with the active comparator BNT162b2 vaccine (Comirnaty^®^, Pfizer-BioNTech) while contributing to characterize the cellular immune response and the long-term duration of antibodies induced by the vaccine candidate.

## Results

### Demographics and baseline clinical characteristics

**Figure 1:**
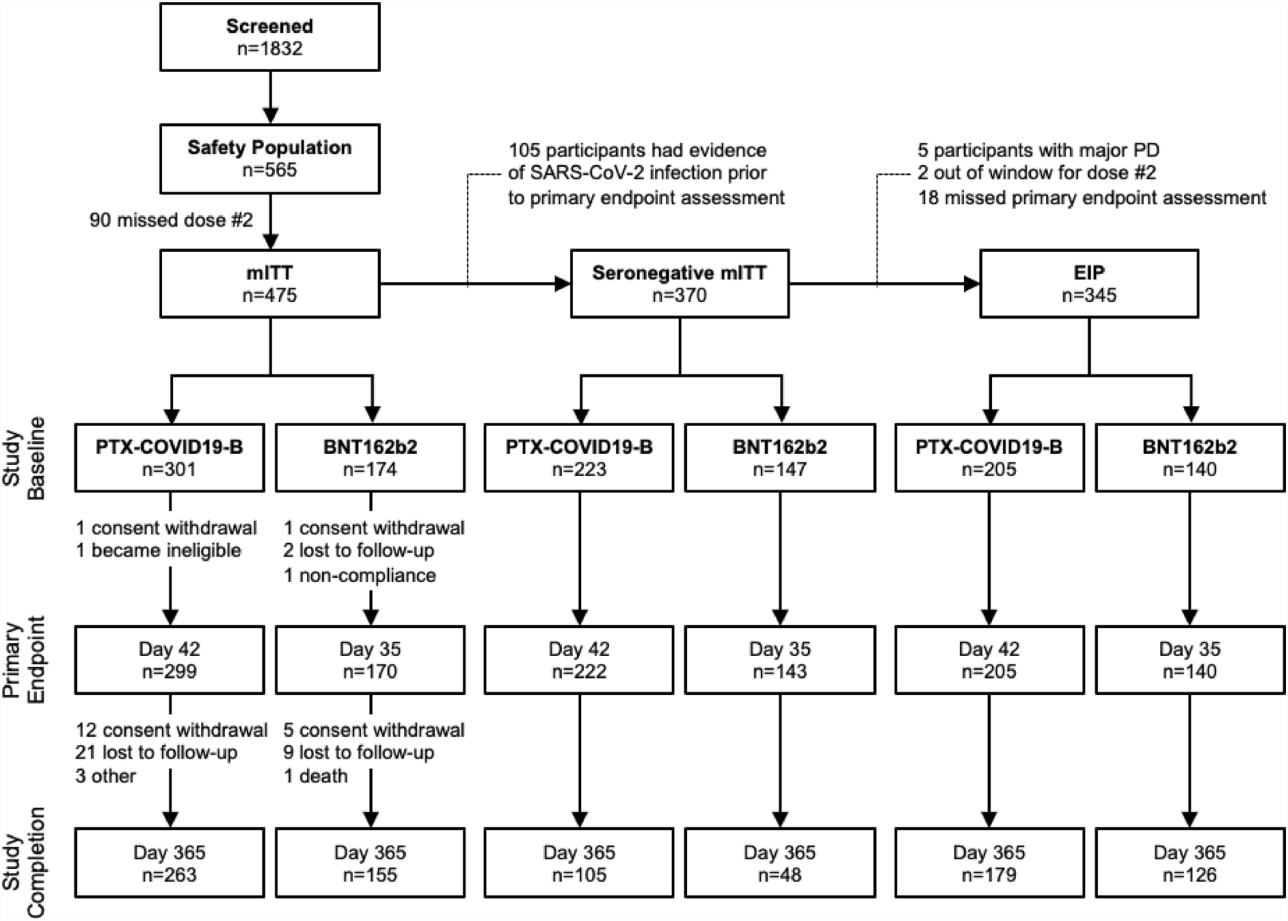
Study profile and participant disposition. Enrollment and follow-up of study participants vaccinated with 40 μg PTX-COVID19-B or 30 µg BNT162b2 after the first and second dose administration. *EIP* evaluable immunogenicity population, *mITT* modified Intent-to-Treat population, *n* number of participants.

Between August 17, 2021 and March 25, 2022, 1832 participants in South Africa and Canada were screened for SARS-CoV-2 antibodies that target nucleocapsid (N) protein. Anti-N seronegative adults 18–64 years (n = 565) were enrolled and randomized 2:1 to receive two doses of 40 µg PTX-COVID19-B or 30 µg BNT162b2, 28 days and 21 days apart, respectively (Fig. 1).

Participant demographics for the Safety Population (i.e. participants who received at least one dose of vaccine) are presented in Table 1. The mean ages for the PTX-COVID19-B (n = 374) and the BNT162b2 (n = 191) groups were 31.4 and 32.3 years, respectively. Participants were predominantly black African in both PTX-COVID19-B and BNT162b2 groups (88.5% and 89.0%, respectively), female representing 47.3% and 48.2% of each group respectively.

**Table 1:** Baseline demographics and clinical characteristics of the Safety Population.

Parameter	Statistics/category	PTX-COVID19-B (n = 374)	BNT162b2 (n = 191)
Age, years	Mean (SD) Min, Max	31.4 (10.31) 18, 64	32.3 (10.90) 18, 64
Gender, n (%)	Male Female	197 (52.7) 177 (47.3)	99 (51.8) 92 (48.2)
Race, n (%)	Black White Other	331 (88.5) 23 (6.2) 20 (5.3)	170 (89.0) 4 (7.3) 7 (3.7)
Ethnicity, n (%)	Non-Hispanic/Latino Other	372 (99.5) 2 (0.5)	190 (99.5) 1 (0.5)

Primary immunogenicity endpoints were measured in the Evaluable Immunogenicity Population (EIP, n = 344). This population excluded participants with evidence of N-binding antibodies in serum or positive rapid S-antigen test confirmed by polymerase chain reaction (PCR) result at any visit prior to the 2-week post second dose blood sample collection. Long-term immunogenicity over 12 months post first dose was investigated in the modified Intent-to-Treat (mITT) population including participants who received the two doses of vaccine and had at least one immunogenicity assessment completed after the initial vaccine dose administration, regardless of serological or virological evidence of SARS-CoV-2 infection. Demographics and baseline clinical characteristics of both EIP and mITT subsets were comparable to the Safety Population (Supplemental Table 1).

### Safety

The primary safety and tolerability endpoints included the incidences of solicited adverse events (AEs) within 7 days after each vaccine dose and occurrence of any AEs from Day 1 (first immunization) through 4 weeks following second vaccination, or Day 57 for participants who missed their second vaccination visit.

#### Solicited systemic and local AEs

**Figure 2:**
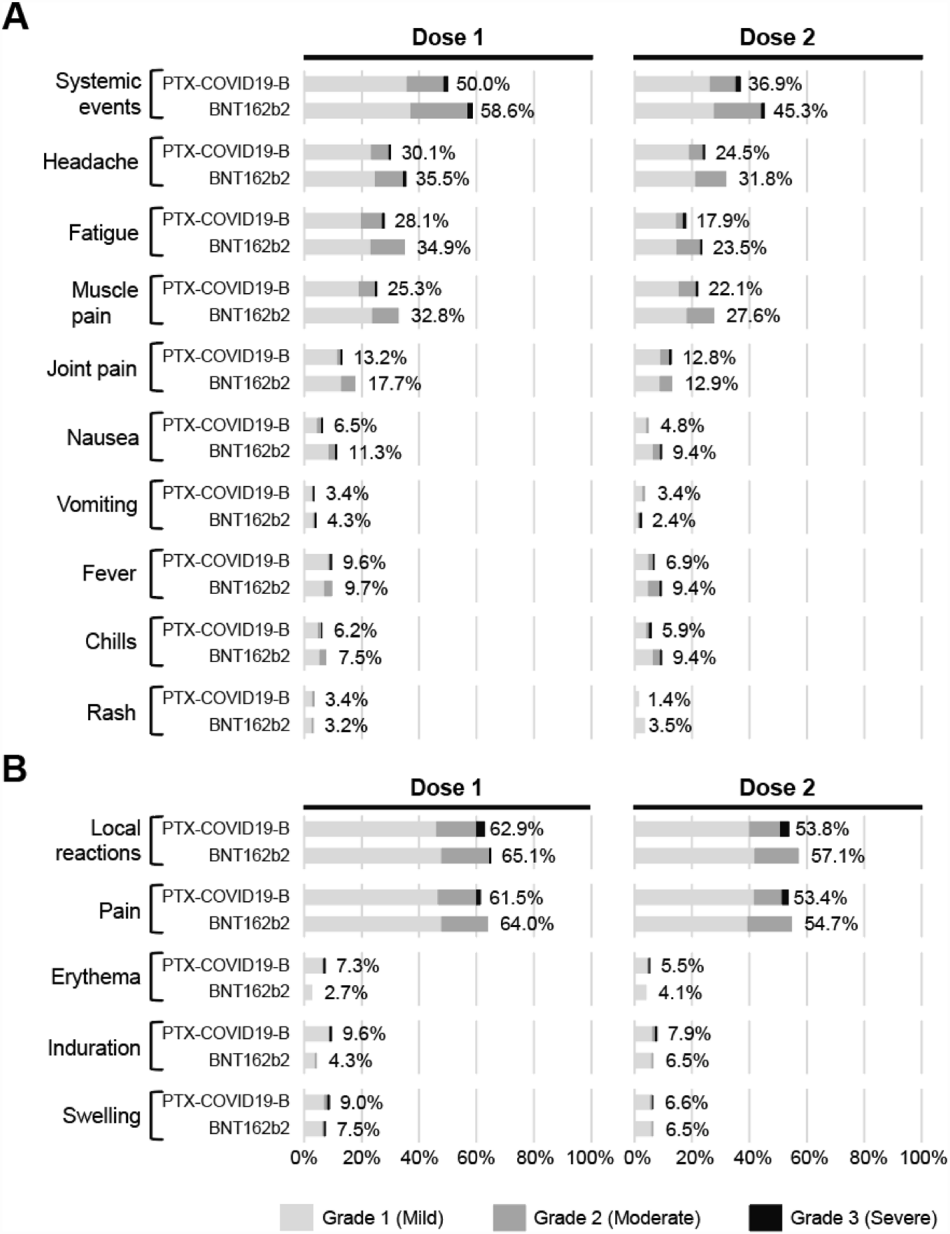
Solicited systemic and local adverse events (AEs) 7 days after each dose. The systemic (**A**) and local (**B**) AEs are reported by dose, treatment and maximum severity. Participants were monitored for solicited systemic and local AEs from the time of vaccination through 7 days after each dose. Occurrence of the events from Grade 1 to Grade 3 are represented as percentage of participants. No Grade 4 (potentially life-threatening) events were reported by participants.

The solicited systemic and local AEs were assessed within 7 days after each vaccine dose (Fig. 2). Solicited systemic AEs were generally lower among participants who received PTX-COVID19-B in comparison to those who received BNT162b2 after each vaccine dose (50.0% vs 58.6% after the first dose and 36.9% vs 45.3% after the second dose). Headache, fatigue, muscle pain, and joint pain were the most common solicited systemic events, reported in ≥ 10% of participants in both treatment groups (Fig. 2A). Participants in the BNT162b2 compared with the PTX-COVID19-B group were more likely to experience headache (35.5% and 31.8% vs 30.1% and 24.5%), fatigue (34.4% and 23.5% vs 28.1% and 17.9%), and muscle pain (32.8% and 27.6% vs 25.3% and 22.1%) after the first and the second dose of the vaccine respectively. Other systemic events, including fever, nausea, chills, rash, and vomiting, were less frequently reported, affecting < 10% of participants in both treatment groups (Fig. 2A). The incidences of solicited local reactions were similar between the treatment groups (Fig. 2B). Pain at the injection site was the most frequent local reaction in both treatment groups, and the incidence was higher after the first vaccine dose compared to the second dose (61.5% vs 53.4% in the PTX-COVID19-B group and 64.0% vs 55.3% in the BNT162b2 group). The incidence of erythema, induration, and swelling at the injection site were low, reported in < 10% of participants overall. All local reactions in both treatment groups were observed within the first 1–2 days after vaccination and resolved shortly thereafter (median: 1–2 days). The overall incidences of solicited local and systemic AEs were more frequently reported after the first dose compared with the second dose of both vaccines and were mostly mild to moderate (Grades 1 or 2) with only < 4% of Grade 3. No Grade 4 reactions were reported (Fig. 2B). Overall, the PTX-COVID19-B vaccine was well-tolerated and no serious AEs leading to death, study discontinuation, events of special interest or potential immune-mediated medical conditions were reported during the two first months after dosing.

#### Unsolicited AEs

The unsolicited AEs (UAEs) were recorded over a period of 12 months and reported in Table 2. Infections (primarily reports of asymptomatic and symptomatic COVID-19) represented the largest portion (63.7%) of reported UAEs. Cumulatively, 442 of 565 participants had UAEs during the study, including 288 participants (77.0%) in the PTX-COVID19-B group and 154 participants (80.6%) in the BNT162b2 group (Table 2). However, UAEs assessed as treatment related were low and only reported in 44 participants (7.8%) with similar proportion between the treatment groups (6.7% and 9.9% in PTX-COVID19-B and BNT162b2 respectively). UAEs assessed as Grade 3 or higher in severity were uncommon with an overall incidence of 3.4%. Serious UAEs were reported in 13 participants, 9 (2.4%) in the PTX-COVID19-B group and 4 (2.1%) in the BNT162b2 group. All serious UAEs were considered unrelated to treatment. Only one participant in each vaccine group reported UAEs leading to study discontinuation. A total of 33 participants (5.8%), representing 6.7% and 4.2% of PTX-COVID19-B and BNT162b2 groups respectively, had medically attended UAEs; 6 of whom (representing 1% of each treatment group) had events considered as new onset of chronic disease. One participant in each vaccine group experienced an event classified as an AE of special interest, but none in either vaccine group had AEs classified as a potential immune-mediated medical condition. One participant in the BNT162b2 group experienced an AE that led to death unrelated to study vaccine. There were no reports of myocarditis or anaphylaxis in the study.

**Table 2:** Summary of unsolicited adverse events (UAEs) over a period of 12 months in the safety population.

	PTX-COVID19-B n = 374	BNT162b2 n = 191	Overall n = 565
Any UAEs, n (%)	288 (77.0)	154 (80.6)	442 (78.2)
Treatment-related UAEs n (%)	25 (6.7)	19 (9.9)	44 (7.8)
Grade ≥ 3 UAEs, n (%)	14 (3.7)	5 (2.6)	19 (3.4)
Treatment-related Grade ≥ 3 UAEs, n (%)	4 (1.1)	1 (0.5)	5 (0.9)
Serious UAEs, n (%)	9 (2.4)	4 (2.1)	13 (2.3)
Treatment-related serious UAEs, n (%)	0 (0.0)	0 (0.0)	0 (0.0)
UAEs leading to study discontinuation, n (%)	1 (0.3)	1 (0.5)	2 (0.4)
Medically attended UAEs, n (%)	25 (6.7)	8 (4.2)	33 (5.8)
New onset chronic disease, n (%)	4 (1.1)	2 (1.0)	6 (1.1)
Adverse event of special interest, n (%)	1 (0.3)	1 (0.5)	2 (0.4)
Potentially immune-mediated medical condition, n (%)	0 (0.0)	0 (0.0)	0 (0.0)
UAEs leading to death, n (%)	0 (0.0)	1 (0.5)	1 (0.2)

### Immunogenicity

#### Humoral responses

**Figure 3:**
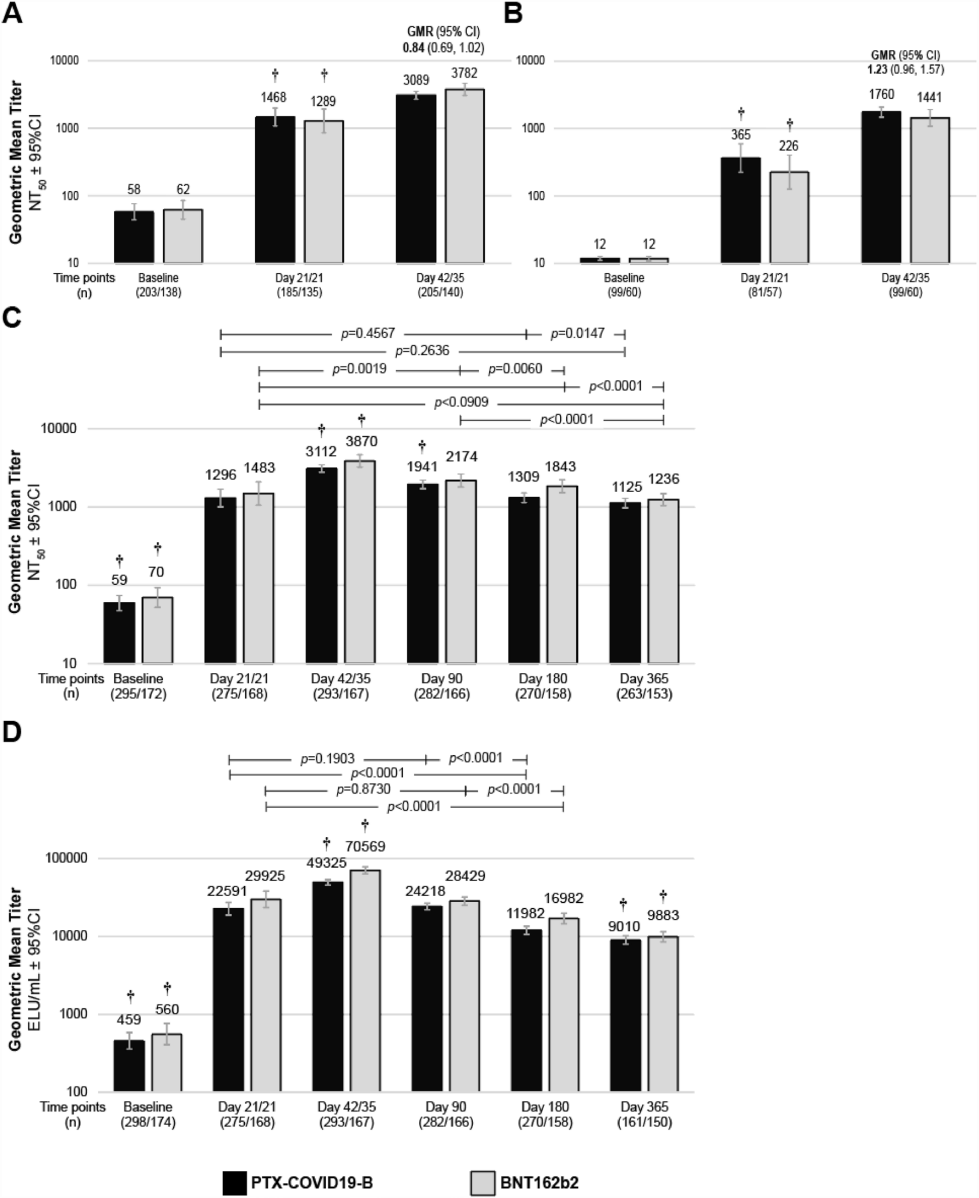
Humoral response. Neutralizing antibody (NAb) titers were measured by ancestral strain derived vesicular stomatitis virus pseudovirus neutralization assay in (**A**) the Evaluable Immunogenicity Population (EIP), (**B**) the EIP with NAb titers ≤ 30 NT50 at baseline and **C**) the modified Intent-to-Treat population (mITT). (**D**) Anti-Spike IgG were measured by ELISA in the mITT population. Geometric mean titers (GMT) including values at the top of the corresponding histograms ± 95% Confidence Interval (CI) represented. The covariance analysis (SAS) of comparing GMT at Day 35/42 in the EIP participants (primary immunological endpoint) are summarized at the top of the corresponding time point histograms (**A**, **B**). † Indicates statistically significant (*p* ≤ 0.0001, paired t-test on log-transformed values, SAS) differences from all other treatment-matched time points. The *p* values for all the other comparisons are presented within group across timepoints (**C**, **D**).

The primary immunogenicity endpoints were measured in 344 EIP participants. Both vaccines elicited a significant (*p* ≤ 0.001) NAb response against ancestral strain 21 days after the first immunization. The second immunization induced a significant (*p* ≤ 0.001) increase of NAb titers 14 days after the boost (Day 35/42) in the EIP (Fig. 3A). On Day 35/42, the NAb geometric mean titer (GMT) and 95% confidence intervals (95%CI) of participants immunized with PTX-COVID19-B (GMT 3068.04, 95%CI: 2675.78, 3517.80) were similar to the titers measured in participants who received two doses of BNT162b2 (GMT 3782.50, 95% CI: 3066.51, 4665.67). The GMT ratio of 0.84 (95% CI: 0.690, 1.017) met the non-inferiority criteria (i.e. the lower bound of the two-sided 95%CI for the GMR comparing treatment groups ≥ 0.67), achieving the primary endpoint of the study. The non-inferiority was also achieved for seroconversion (i.e. achieving a ≥ four-fold increase over pre-vaccination at Day 35/42) with 89.0% and 92.7% of the participants who seroconverted in the PTX-COVID19-B and BNT162b2 groups respectively (Table 3). Despite exclusion of anti-N seropositive participants from the EIP, relatively high NAb titers were observed at baseline for several participants across both treatment groups. Presence of substantial anti-S antibodies in anti-N seronegative adults have been reported^1,17^ and could result from previous (asymptomatic) SARS-CoV-2 infection compounded by NAb and anti-N differential kinetics^18,19^. Differences in anti-S and anti-N assay sensitivities and different half-life for the antibodies, including NAb, could also have contributed to the observed NAb values at baseline in a small subset of participants in EIP^20^. In an additional post-hoc analysis excluding participants with NAb titers > 30 NT_50_ (i.e. three times the Lower Limit of Detection) at baseline, the seroresponse rates 2 weeks after the second dose reached 100% for both treatment groups. The non-inferiority of the NAb response was also confirmed in the EIP subjects with NAb titers ≤ 30 NT_50_ at baseline (Fig. 3B). The long-term duration of antibodies after the administration of the first dose of PTX-COVID19-B and BNT162b2 vaccines was assessed over 12 months. Due to the surge of infections caused by the emergence of the Omicron BA.1 VOC (‘Omicron wave’) in South Africa during this phase 2 clinical study, the durability of the humoral response was assessed on the mITT population. Both vaccines elicited a significant (*p* ≤ 0.001) NAb response 21 days after the first immunization in the population which included both anti-N seropositive and seronegative participants (GMT 1295.76, 95% CI: 1002.44, 1674.91 and GMT 1483.24, 95% CI: 1053.20, 2088.87 for PTX-COVID19-B and BNT162b2 groups respectively). As observed with anti-N seronegative participants from the EIP, the NAb titers further significantly (*p* ≤ 0.001) increased 14 days after the boost in both treatment groups (Fig. 3C). NAb titers peaked 2 weeks after the second dose on Day 35/42 and significantly (*p* ≤ 0.001) decreased over time similarly in both treatment groups. By Day 90, GMT were approximately 38% and 44% lower relative to Day 35/42 for PTX-COVID19-B and BNT162b2, respectively. Although experiencing a relative decrease from Day 35/42 (64% and 68% for PTX-COVID19-B and BNT162b2, respectively) NAb GMT remained significantly (*p* ≤ 0.001) higher than baseline and comparable to Day 21/21 1 year after receiving the first immunization (Fig. 3C). The NAb titers measured in participants without evidence of SARS-CoV-2 infection during the study were lower compared to those for whom infection likely acted as a ‘natural booster’. That difference became statistically significant (*p* ≤ 0.05) at Day 90 in participants who received the PTX-COVID19-B vaccine candidate while for the BNT162b2 group statistical significance was observed at Day 365 (Supplementary Fig. 1). The durability of the immunoglobulin G (IgG) responses was also examined on the mITT population. Both vaccines induced a significant (*p* ≤ 0.001) increase of anti-S binding IgG titers after the first (Day 21) and the second immunization (Day 35/42). Despite experiencing a significant (*p* ≤ 0.001) decrease on Day 90 (60% vs 51% decline from Day 35/42 for BNT162b2 and PTX-COVID19-B respectively), IgG titers remained significantly higher than baseline values 1 year after receiving the first immunization (Fig. 3D).

**Table 3:** Seroresponse rate based on neutralizing antibody titers for ancestral SARS-CoV-2 in the Evaluable Immunogenicity Population.

	PTX-COVID19-B n = 205	BNT162b2 n = 140
Seroresponse rate at day 35/42, %	89.11	92.75
Seroresponse Rate 95% CI^[1]^	83.98, 93.05	87.08, 96.47
Difference in seroresponse rates (95% CI)^[2]^	− 3.64 (− 9.77, 2.96)	

Seroconversion was defined as four-fold or greater increase in SARS-CoV-2-specific neutralizing antibody titres between Day 1 and post-vaccination sample collection time points. CI = confidence interval; pre-specified non-inferiority margin of difference in seroresponse rate = − 10.0%

^[1]^Clopper-Pearson confidence interval.

^[2]^2-sided Miettinen and Nurminen confidence interval.

#### Cell-mediated immunity

**Figure 4:**
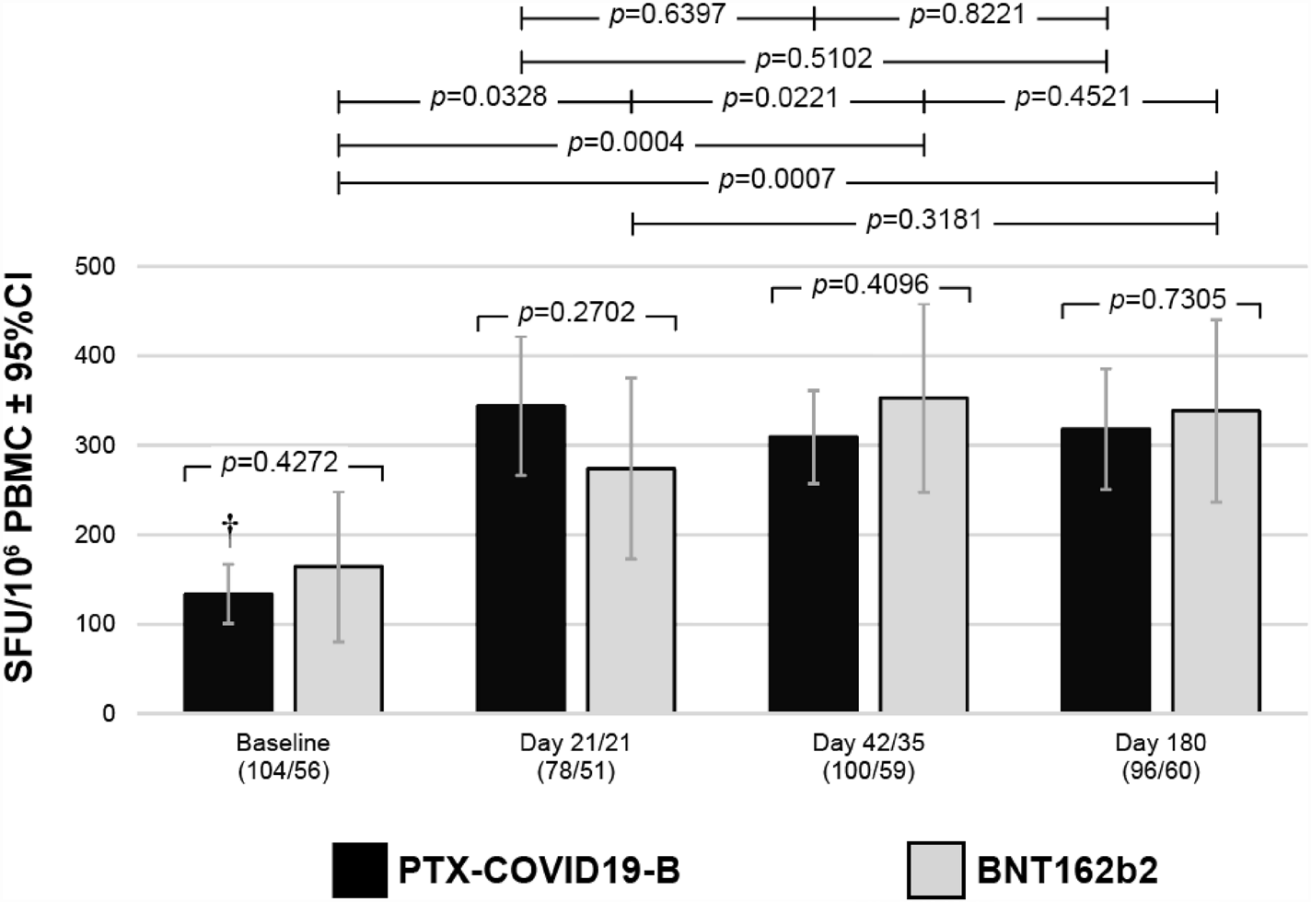
Cell-mediated immunity. IFN-γ response was measured in the modified Intent-to-Treat population by ELISpot. Mean of Spot Forming Cell (SFC) counts per 10^6^ cells ± 95% Confidence Interval (CI) are represented. † Indicates statistically significant (*p* ≤ 0.0001) differences from all other treatment-matched time. The *p* values for all the other comparisons are presented as within group values at each timepoint or between group values across timepoints (paired t-test on log-transformed values, SAS).

The interferon-γ (IFN-γ) and interleukin-5 (IL-5) S-specific cellular immune responses were measured by enzyme-linked immunosorbent spot (ELISpot) in peripheral blood mononuclear cells (PBMC) from both mITT and EIP participants. The IFN-γ responses observed in the two populations were similar. Both vaccines elicited a significant IFN-γ response as soon as 21 days after the first dose. While the number of S-specific IFN-γ secreting cells peaked after the first dose in participants immunized with a single dose of PTX-COVID19-B, the response was more gradual in the BNT162b2 group with a significant increase observed between the first and the second dose. However, no difference between treatment groups were observed at any time points and both vaccines induced a durable IFN-γ response up to 6 months after the first immunization (Fig. 4). Limited-to-no induction of S-specific IL-5 response was observed in both groups along the study period (data not shown).

#### Mucosal immune response

**Figure 5:**
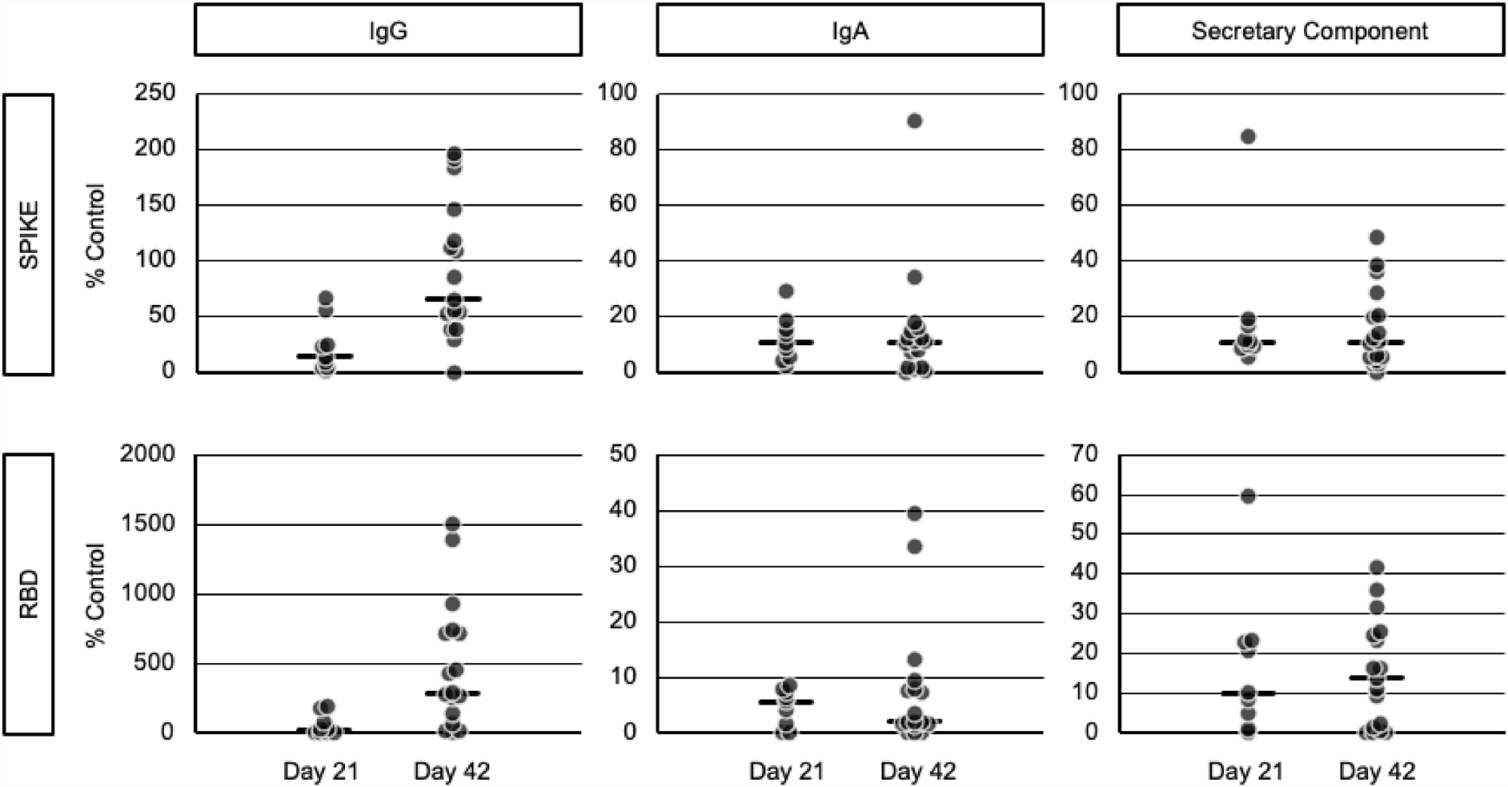
Mucosal immunity. Anti-Spike and anti-Receptor Binding Domain (RBD) IgG, IgA and associated secretory component were measured by ELISA at serial dilutions in 17 participants (mITT). The Area Under the Curve (AUC) was then normalized and expressed as % of internal positive control consisting of saliva collected from COVID-19 acute and convalescent patients.

Vaccine inducing mucosal immunity at the site of infection have the potential to prevent virus infection, replication, and shedding and therefore, transmission^21^. The S-specific IgG, IgA and secretory component (SC) were measured in saliva from a limited number of PTX-COVID19-B participants. These preliminary results revealed the induction of S-specific mucosal response after one (Day 21) and two (Day 42) doses of PTX-COVID19-B vaccine (Fig. 5). While levels of IgG in saliva appeared to benefit of a second immunization, the IgA and SC remained unchanged between Day 21 and Day 42 (Fig. 5).

### Preliminary efficacy assessment

**Figure 6:**
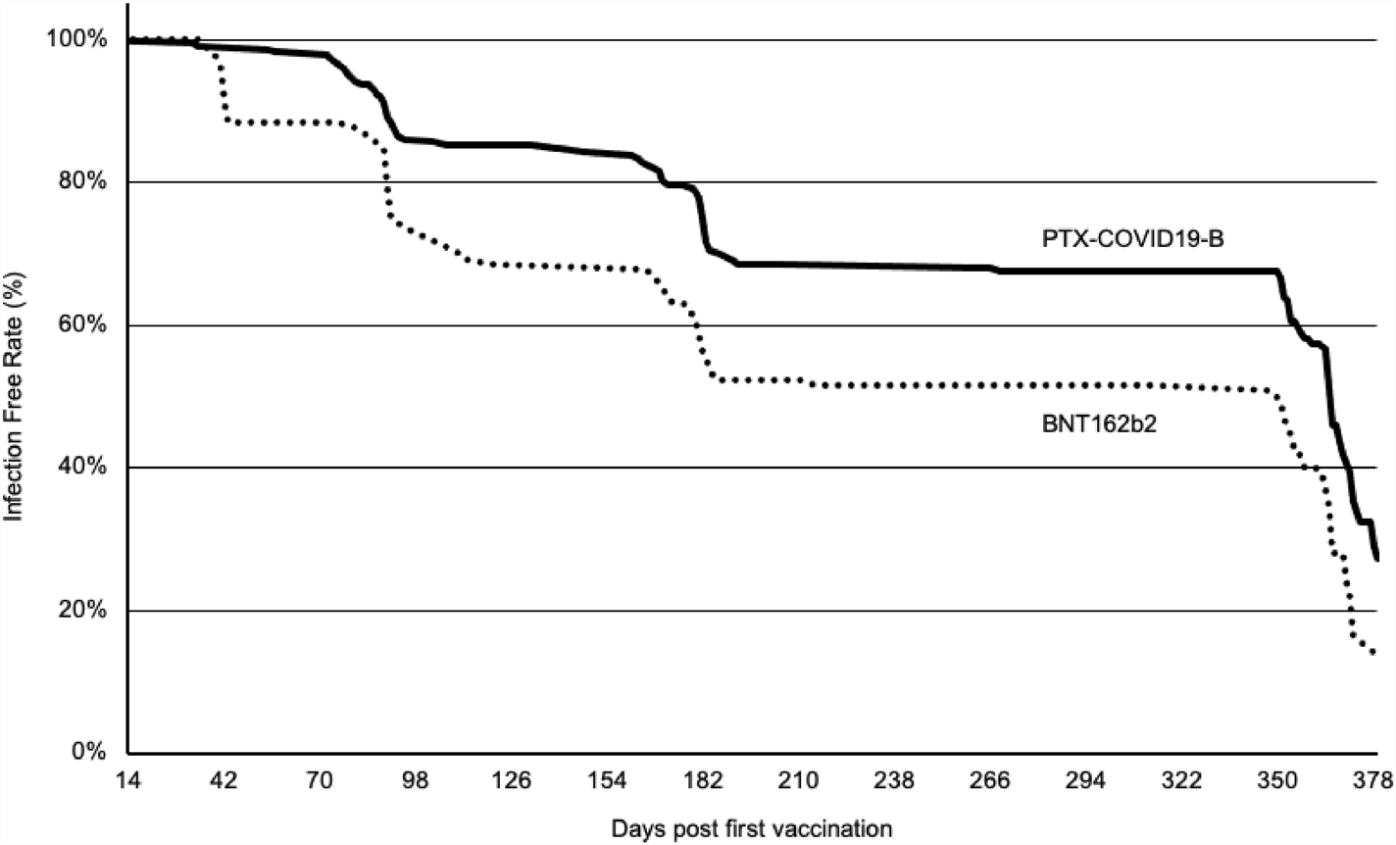
Time to COVID-19 infection. Cumulative incidence of COVID-19 infection free participants in seronegative modified Intent-to-Treat population starting 2 weeks after the first vaccine dose. COVID-19 cases were confirmed by PCR-positive nasopharyngeal swabs and/or reactive serum antibody test results.

Due to the Omicron wave during the study, over 50% of participants in both vaccine mITT populations experienced breakthrough infection (53.2% and 67.3% in PTX-COVID19-B and BNT162b2 respectively), allowing for a post-hoc analysis of the relative effectiveness of the vaccines over the 1-year duration of the study (Fig. 6). We specifically considered cases of SARS-CoV-2 infection occurring 2 weeks after the first vaccine dose, excluding those infected earlier from our analysis. The infection was confirmed by serological and virological testing. No severe cases or hospitalization were observed in any of the vaccine groups. Notably, subjects vaccinated with PTX-COVID19-B exhibited a significantly (*p* = 0.0002) lower incidence of infections compared to those who received the active control vaccine BNT162b2 at every investigated time points (Table 4).

**Table 4:** Kaplan–Meier estimates of the participants remaining COVID-19 uninfected in the seronegative modified intend-to-treat population.

Time points (post first dose)	PTX-COVID19-B (n = 223) SDF % (95% CI)	BNT162b2 (n = 147) SDF % (95% CI)
2 months	98.2 (95.3, 99.3)	88.4 (81.9, 92.6)
3 months	85.6 (80.3, 89.6)	73.5 (65.5, 80.0)
6 months	68.5 (61.8, 74.2)	52.2 (43.7, 60.0)
12 months	27.3 (18.7, 36.6)	13.7 (6.1, 24.4)
Log-rank test	*p* = 0.0002	

Kaplan–Meier estimates from product-limit method at the end of visit windows. Confidence intervals constructed using log–log transformation.

*SDF* survival distribution function.

## Discussion

In this phase 2 immunobridging clinical study, we compared the safety, tolerability and immunogenicity of PTX-COVID19-B vaccine candidate with the active-control, authorized BNT162b2 vaccine that demonstrated high efficacy in clinical studies^3^. Both mRNA vaccines demonstrated similar safety and tolerability profiles, consistent with previously reported clinical studies with mRNA vaccines^2,3,22^. There were no reports of myocarditis or anaphylaxis, which, although rare, are known potential side effects of mRNA-based vaccines^23,24^. Our primary goal was to compare the NAb GMT and seroconversion rates 2 weeks after the second dose between these two vaccines. Importantly, our analysis met the non-inferiority criteria, successfully achieving the primary immunological endpoints set for this phase 2 study. Two doses of the PTX-COVID19-B vaccine elicited a rapid induction of humoral response followed by relatively slow waning with approximately 58% and 75% lower NAb and IgG antibodies, respectively, 6 months after the second dose. The non-inferiority of NAb response was maintained after 1 year in a mixed population including participants with breakthrough cases. Although BNT162b2 elicited higher levels of IgG antibodies at the peak of the response 2 weeks after the second dose, these levels declined more rapidly in that group, resulting in IgG titers of similar amplitude between PTX-COVID19-B and BNT162b2 1 year after the initial immunization. Differential kinetics between NAb and anti-S IgG has been previously reported^25^. While NAb have been demonstrated as a correlate of protection for COVID-19^6,8^, accumulating data also support association between binding antibodies and vaccine efficacy across diverse vaccine platforms^7,26^. Moreover, differential impact of the different administration timing of the second dose on the IgG and NAb kinetics cannot be ruled out, particularly in pre-infected individuals for who anamnestic response would have been primed by the pre-infection.

Despite EIP excluding anti-N seropositive participants as marker of previous SARS-CoV-2 infection, relatively high NAb titers were observed at baseline in several participants in both treatment groups. Presence of substantial anti-S IgG and/or NAb titers in anti-N seronegative participants have been previously reported^17,27^ and could result from previous (asymptomatic) SARS-CoV-2 infection compounded by NAb and anti-N differential kinetics^18–20^. Differences in assay sensitivity as well as cross-reactive neutralizing antibodies resulting from previous endemic coronavirus infections could also have contributed to high NAb values at baseline. The lowest NAb response was observed in participants in both groups with NAb titers < 30 NT_50_ at baseline and no breakthrough infection, while participants who experienced breakthrough infection during the study displayed the higher NAb GMT titers. When compared to the peak antibody response, a significant decline 3 months post-vaccination with PTX-COVID19-B and BNT162b2 vaccines was reported in the population that included both anti-N seropositive and seronegative participants. Nonetheless, participants with breakthrough omicron SARS-CoV-2 infection during the 12-month follow-up displayed higher NAb titers in both treatment groups. This illustrated the previously reported beneficial impact of hybrid immunity after vaccination^28,29^. Formation of memory B cells by vaccination and robust enhancement of serologic responses by SARS-CoV-2 infection results in durable^30^, high levels of cross-variant neutralizing antibodies^31^ and reduces the risk of reinfection^32^. Both vaccines therefore appeared to induce similarly memory B cell priming in germinal center which can be remobilized to produce a robust anamnestic response during infection. In addition to the humoral response, COVID19-B mRNA vaccines have demonstrated their ability to induce strong cell-mediated immunity (CMI). In this phase 2 study, S-specific IFN-γ and IL-5 ELISpot responses were measured in a relatively large, randomly selected, participant population compared to previous clinical trials assessing the cellular immune response^33,34^. As previously reported, a minority of participants in each group had substantial pre-existing IFN-γ response to S protein, likely resulting from previous exposure to either endemic and/or pandemic coronaviruses^35,36^. The PTX-COVID19-B vaccine candidate and BNT162b2 induced similar levels of S-specific IFN-γ producing cells in agreement with the responses previously reported for BNT162b2^28,37^. Cellular immunity is required for recovery from almost all viral infections and for the maintenance of long-term protection^38,39^. Coordinated early activation of cell-mediated and humoral immunity was also associated with reduced severity of COVID-19^40,41^. Moreover, CMI is intrinsically more cross-reactive than humoral immunity^42–44^, an increasingly important consideration with the continued emergence of SARS-CoV-2 variants^45^. As opposed to NAb displaying significant decrease in neutralizing activity against VOC, the T cell response induced by newly developed mRNA vaccines was generated against conserved epitopes with limited-to-none impact of point mutations^44,46^. Consequently, Th1 driven T-cell response has been proposed to play a pivotal role in controlling the disease and vaccine provided cross-protection against severe disease with low NAb titers against VOC^47^. Even though a small population was evaluated in relative effectiveness, PTX-COVID19-B showed a degree of preventing ongoing Omicron BA.1 infections in the South African population analyzed in this study. The induction of IL-5 was associated with detrimental outcomes, inappropriate inflammation and immunologically related vaccine serious adverse events during coronavirus infections^48,49^. The absence of IL-5 induction is consistent with the safety profiles. In addition to the humoral and cell-mediated immune responses, early data in a limited number of participants suggest that PTX-COVID19-B may have a potential to -induce mucosal immunity. Anti-S and anti-RBD IgG and IgA have been previously measured in saliva after two intramuscular (IM) injections of BNT162b2 and could be associated with protection against infection considering the role of mucosal immunity at the site of infection to prevent virus infection and replication^50^. During the COVID-19 pandemic, the mRNA vaccine platforms have emerged as powerful tools in our arsenal against infectious diseases offering rapid development timelines, efficient production processes, and a remarkable safety profile. Their ability to swiftly adapt to the ever-evolving landscape of novel SARS-CoV-2 VOCs demonstrated their remarkable versatility, flexibility, and agility enabling us to respond effectively to emerging threats. The PTX-COVID19-B vaccine candidate has demonstrated acceptable and comparable safety as well as non-inferior and durable humoral immune response and similar CMI compared to the active comparator BNT162b2 vaccine. Globally the SARS-CoV2 has evolved towards more distant Omicron variants. In this context, Providence Therapeutics mRNA platform supports acceleration of next-generation COVID-19 vaccine candidates.

## Methods

### Vaccine candidate and dose selection

PTX-COVID19-B is a mRNA-based vaccine composed of a lipid nanoparticle containing modified mRNA that encodes for the full-length S protein from SARS-CoV-2 with amino acid glycine at position 614 (G614) and is > 95% identical to the antigens encoded by the existing authorized mRNA vaccines BNT162b2 (Comirnaty^®^, Pfizer) and mRNA-1273 (Spikevax^®^, Moderna). The sequence in PTX-COVID19-B does not contain any proline substitutions in S2 domain to stabilize the pre-fusion S protein as previously described^16,51^. The dose was selected based on data from the Phase 1 Study^16^, which explored dose levels of 16, 40, and 100 μg PTX-COVID19-B. The 40 μg dose was safe, well tolerated with reduced local site reactions when compared to the 100 μg dose. Two doses of 40 µg displayed the right balance between the immune response and satisfactory reactogenicity profile providing an immune response that was comparable to the immunogenicity observed for other mRNA vaccines and was therefore selected for evaluation in this phase 2 clinical study.

### Study design

This observer-blind, double-dummy, randomized phase 2 study (NCT05175742, 04/01/2022) was conducted at five centers in Canada and five centers in South Africa. Enrolment started in August 2021. The study was approved by the Wits University Human Research Ethics Committee, the University of Cape Town Human Research Ethics Committee (South Africa) and the Advarra Central Institutional Review Boards (Canada). All research was performed in accordance with relevant guidelines/regulations. Adults 18–64 years of age were screened for the presence of SARS-CoV-2 nucleocapsid (N) IgG antibodies using commercial immunoassays accordingly to the manufacturer’s instructions (ElecSys, Roche Diagnostics, Canada or Alinity, Abbott, South Africa). All participants enrolled voluntarily and provided written informed consent before any study procedures. Only N-seronegative participants (n = 565) were subsequently randomized, via an Interactive Web Response System (IWRS) within the electronic data capture (EDC) system using a randomization schedule produced with SAS software v9.4 or higher, 2:1 to receive intramuscular (IM) injection in deltoid muscle of either 40 μg PTX-COVID19-B (n = 374) or 30 µg BNT162b2 (n = 191), in a blinded manner. Exclusion criteria are detailed in Supplemental Material. Participants assigned to the BNT162b2 treatment arm received their second dose 21 days after first immunization (Day 21), and participants assigned to the PTX-COVID19-B treatment arm received their second dose on Day 28. To preserve the observer-blind (including treatment assignment), a double-dummy design was used where placebo was administered to the relevant treatment arm at each of the dosing visits. Participants, vaccine administrators, and clinical staff performing evaluations were blinded to treatment. Clinical staff involved in the preparation of the vaccine or placebo were aware of what the participant received and prepared these out of sight of all other staff and the participant.

### Safety and reactogenicity assessments

Clinical and laboratory evidence of AEs were monitored for each participant on a routine basis throughout the study including the date of onset, description, severity, duration, relationship of the AE to the investigational study drug, action(s) taken and outcome. Solicited AEs including reactogenicity events were reported through the seventh day after each vaccine dose administration and graded 1 to 4 (mild, moderate, severe, or potentially life-threatening) according to criteria described in the Protocol (Supplementary Material). The UAEs were recorded over a period of 12 months. Safety data after each dose were reviewed by the Independent Data and Safety Monitoring Board (DSMB) according to the specific responsibilities and activities defined in the DSMB Charter (Protocol, Supplemental Material). Throughout the study, the Pharmacovigilance team at Providence Therapeutics were reviewing AEs of special interest and serious AEs.

### Immunogenicity assessments

#### Pseudovirus neutralizing antibody response against ancestral strain

Neutralizing antibodies were measured using a validated pseudovirus neutralization assay by Nexelis (QC, Canada) as previously described^16,52^. Briefly, pseudotyped virus particles were generated from a modified Vesicular Stomatitis Virus (VSVΔG) backbone and bore the SARS-CoV-2 S protein from which the last 19 amino acids of the cytoplasmic tail were removed. Seven two-fold serial dilutions of the heat-inactivated human serum samples were prepared in duplicates in 96-well plates. The pseudovirus were added sequentially to the serum dilutions at a target working dilution and incubated at 37 °C with 5% CO_2_ supplementation for 60 ± 5 min. Serum-virus complexes are then transferred onto plates previously seeded overnight with Vero E6 cells (ECACC, Cat No 85020206) expressing ACE-2 receptor and incubated at 37 °C and 5% CO_2_ for 20 ± 2 h. Cells were then lysed, and ONE-GLO luciferase reagent (Promega, Madison, WI) was added for 2 min prior to reading with SpectraMax i3x plate reader (Molecular Devices, San Jose, CA). The intensity of the luminescence was quantified in relative luminescence units (RLU, SoftMax Pro software) and is inversely proportional to the level of neutralizing antibodies present in the serum. The neutralizing titer of a serum sample is calculated as the reciprocal serum dilution corresponding to the 50% neutralization antibody titer (NT_50_).

#### Spike specific IgG ELISA

A validated (Nexelis, QC, Canada) indirect ELISA was performed to measure S specific IgG as previously described^16^. Briefly, The SARS-CoV-2 pre-fusion S antigen was adsorbed onto a 96-well microplate and standard ELISA procedure was followed with anti-SARS-CoV-2 S IgG specific antibody (primary antibody) and antihuman IgG antibody (secondary antibody) conjugated to peroxidase. The absorbance was measured using a spectrophotometer at 450/620 nm. A standard on each tested plate was used to calculate the SARS-CoV-2 S binding IgG according to the unit assigned by the standard (ELU/mL).

#### Spike specific IL-5 and IFN-γ ELISpot

Extra blood samples were collected in sodium heparin (NaHep) tubes from the first 90 subjects upon consent agreement (Protocol, Additional Information) and sent directly to the University Health Network (Toronto, Canada) or at the Bio Analytical Research Corporation (Johannesburg, South Africa) for PBMC isolation, cryopreservation and storage. Samples were then transported to Nexelis (Laval, QC, Canada) to perform validated double color IFN-γ and IL-5 enzymatic enzyme linked immunosorbent spot (ELISpot) assay using Cellular Technology Limited (CTL, OH, USA) kit. Briefly, 96-well polyvinylidene difluoride (PVDF) plates were coated overnight with anti-human IFN-γ/IL-5 capture antibodies. Following the overnight coating, PBMC (2 × 10^5^ cells/well) were incubated in the plates with peptide pools (1 µg/mL, purity > 70%, JPT Peptides) covering the full sequence of SARS-CoV-2 S protein (Uniprot ID P0DTC2), dimethyl sulfoxide (DMSO, negative control) or Phytohemaglutinnin (PHA, positive control) in triplicates. After 44 ± 1 h incubation at 37 °C with 5% CO_2_, plates were washed twice with D-PBS followed by two additional washes with PBS-0.05% Tween. The anti-human IFN-γ/IL-5 Detection Solution containing Anti-human IFN-γ (Fluorescein isothiocyanate (FITC) and Anti-human IL-5 (Biotin) detection antibodies was then added to the wells. Following another round of three washes with PBS-0.05% Tween, FITC-Horseradish peroxidase (HRP) and Streptavidin–Alkaline Phosphatase (ALP) were added, and plates were incubated 60 ± 10 min at room temperature protected from sunlight. The plates were washed two times PBS-0.05% Tween followed by two times with Milli-Q Water before the addition of Blue and Red Developer Solutions in sequence (with washes in between the two Developer solutions) accordingly to the manufacturer instructions. The blue (IL-5) and red (IFN-γ) spots indicating the presence of IL-5 and IFN-γ producing cells (i.e. spot forming unit, SFU) were numbered using the ImmunoSpot CTL Analyzer and expressed as number of SFU per million of cells (SFU/10^6^ cells). Reported values were presented as DMSO subtracted values i.e. the mean of the negative control duplicates was subtracted from the mean of the SARS-CoV-2 S protein stimulated cells.

#### Mucosal immunity in saliva

S and RBD specific IgG, IgA and secretory component in saliva were measured as previously described^50^ in 17 participants from mITT. Saliva samples were collected using Salivette^®^ tubes (Sarstedt, Numbrecht, Germany), a collection system which consists of a cotton ball which participants chew for exactly 3 min and place into a tube, which was then placed into a larger outer tube. The entire system was centrifugated (1000×*g*) for 5 min at room temperature. The total saliva volume from each participant was then stored at − 80 °C until the time of testing. The biotinylated S, RBD or phosphate-buffered saline solution (PBS, negative control) were added to streptavidin pre-coated 96-well plates and incubated overnight at 4 °C. The coating solution was then discarded and plates were blocked with 5% (w/vol skim milk powder) BLOTTO solution (BioShop, ON, Canada) at 37 °C for 2 h. Sample dilutions in 2.5% BLOTTO (1:5–1:20) were pre-incubated in a separate streptavidin-coated plate with no antigen to reduce anti-streptavidin activity in the saliva before addition to the coated plates and incubated 2 h at 37 °C. The antigen-coated plates were then washed 3 times with PBS + 0.05% Tween 20 before the addition of Horse radish peroxidase (HRP)-conjugated goat anti-human-IgG and IgA secondary antibodies (Southern Biotech, IgG: 2044-05, IgA: 2053-05) and incubated for 1 h at 37 °C. The substrate Solution 3,3′,5,5′tetra-methylbenzidine (TMB, ThermoFisher) was then added to each well and the reaction was then stopped by addition of 1 N H_2_SO_4_. The optical density was read at a wavelength of 450 nm (OD450) on a spectrophotometer (Thermo Multiskan FC). The OD450 for the PBS control was subtracted from the antigen-specific OD450 value for each sample, at each sample dilution and adjusted OD450 value was used to calculate the area under the curve (AUC). The sample AUC was then normalized to the AUC of the positive control, which consisted of saliva collected from COVID-19 acute and convalescent participants. The normalized AUC was multiplied by 100 to give a final percentage, which we deemed the “% of positive control”^50^. Secretory component-associated antibodies were detected by modifying our saliva Spike/RBD ELISA by using an HRP-conjugated Goat anti-human secretory component detection reagent at a dilution of 1:750 from Nordic MUBio (Netherlands).

### Preliminary efficacy assessment

The incidence of COVID-19 infections occurring 14 days after the first dose was monitored in the seronegative (anti-N IgG) mITT population. COVID-19 infections were reported as adverse events after positive rapid S-antigen tests performed at the clinical site or at home and were confirmed by positive PCR test and/or reactive serum antibody test results. Reported symptoms of COVID-19 were also confirmed as COVID-19 infections by positive PCR test and/or reactive serum antibody test results. Time to COVID-19 infection was analyzed using Kaplan–Meier product limit methods to compute 25th, 50th (median) and 75th percentiles with associated two-sided 95%CI, as well as percentage of censored observations. Observations were censored at earliest available date representing early termination, last contact, or end of study. Kaplan–Meier estimates time of COVID-19 infection from Survival Distribution Function were calculated with associated confidence intervals at 2, 3, 6 and 12 months. A log-rank *p*-value for comparison of PTX-COVID19-B and BNT162b2 vaccine groups were computed to assess differences in Kaplan–Meier curves.

### Statistical analysis

This phase 2 study was designed to provide estimates of safety and immunogenicity for 40 μg PTX-COVID19-B in comparison to BNT162b2 (Comirnaty^®^, Pfizer-BioNTech) COVID-19 vaccine. A total sample of 565 participants were enrolled and randomized 2:1 (374 to PTX-COVID19-B and 191 to BNT162b2). The sample size was selected to allow sufficient likelihood of observing AEs that occur even at low rates, as were expected in this study as following the Center for Disease Control and Prevention guidelines (2021) as detailed in the Study Protocol (Supplemental material). All baseline, safety, and tolerability analyses were assessed on the Safety Population, which included all participants who received any amount of vaccine. The primary immunological endpoints posited the non-inferiority of the NAb GMT induced by the vaccine candidate compared to the response induced by the active comparator. According to regulatory guidance and recommendation from the FDA and the European Medicines Agency, non-inferiority in NAb GMT can be established if the lower boundary of a two-sided 95% CI surrounding the ratio of the GMT for the vaccine candidate vs the active comparator does not fall below 0.67. Equivalently, the lower boundary of a two-sided 95% CI surrounding the mean difference between the log (GMT) must not fall below − 0.176. The standard deviation (SD) of the NAb GMT for BNT162b2 was derived from 95% CIs reported in literature, ranging from 0.16 to 0.26 (logarithmic scale). Assuming a conservative value of 0.26, with a one-sided type I error rate of 0.025, 90% power, and assuming a 2:1 randomization, a minimum of 71 participants enrolled to PTX-COVID19-B versus 36 participants to active comparator was required. The NAb titers were analyzed on the logarithmic (log10) scale and reported for baseline and Day 35/42 comparisons using an analysis of covariance (ANCOVA) model with baseline value, age, and treatment arm as explanatory variables. Least square (LS) means (on log10 scale), standard error, and GMT were computed for each study arm and visit. The difference between study arms were compared at Day 35/42 using LS means and are presented on the logarithmic scale and exponentiated with corresponding 95% CI. The frequency and percent of participants with seroresponse are summarized with 95% Clopper Pearson CI at Day 35/42. The difference in percentages between PTX-COVID19-B and BNT162b2 was computed with the associated two-sided 95% Miettinen and Nurminen CI. Baseline demographics and safety were measured on the Safety Population which included all participants who received at least one dose of vaccine. The primary immunological endpoints were assessed on EIP which included participants without evidence of SARS-CoV-2 infection before or during vaccination assessment period, who received both doses of vaccine and had post-baseline immunogenicity measurement(s). Occurrence of SARS-CoV-2 infections were monitored by serological or virological assays (i.e., N-binding antibody [serum] or negative polymerase chain reaction result) at any visit prior to the 2-week post second dose blood sample collection. Persistence of long-term memory immune response was measured on the mITT population including all randomized participants who received both vaccinations and had at least one immunogenicity assessment completed after the initial vaccine dose administration. The statistical analysis and data presentation were conducted using SAS software, v9.4 or higher (SAS Institute, North Carolina).

### Supplementary Information

Supplementary Information.

## Data Availability

The datasets associated with this study are available from the corresponding author upon request. The Protocol and Statistical Analysis Plan are provided as supplementary materials. The study is registered on ClinicalTrials.gov under the identifier NCT05175742.
