# Supplementary material for: Comparable safety and non-inferior immunogenicity of the SARS-CoV-2 mRNA vaccine candidate PTX-COVID19-B and BNT162b2 in a phase 2 randomized, observer-blinded study
Source: Sci Rep. 2024 Mar 4;14:5365. doi: 10.1038/s41598-024-55320-1 (PMC10912344; doi:10.1038/s41598-024-55320-1)

**Supplementary Figure 1**: **Impact of breakthrough infection on neutralizing antibody response.** Comparisons between neutralizing antibody titers measured by ancestral strain derived vesicular stomatitis virus pseudovirus neutralization assay in modified Intent-to-Treat participants with vs without breakthrough infection. Geometric mean titers including values at the top of the corresponding histograms ± 95% Confidence Interval (CI) and *p* values (SAS) are represented.

**Supplementary Figure 2: Cell-mediated immunity.** IL-5 response was measured in the modified Intent-to-Treat population by ELISpot. Mean of Spot Forming Cell (SFC) counts per 10^6^ cells ± 95% Confidence Interval (CI) are represented.

**Supplementary Table 1: Baseline demographics and clinical characteristics of the Evaluable Immunogenicity Population and Modified Intent-to-treat Population.**

|  | | **Evaluable immunogenicity** | | **Modified Intent-to-Treat** | |
| --- | --- | --- | --- | --- | --- |
| **Parameters** | **Statistics**  **Category** | **PTX-COVID19-B** (n = 205) | **BNT162b2** (n = 140) | **PTX-COVID19-B** (n = 301) | **BNT162b2** (n = 174) |
| Age, years | Mean (SD) Min, Max | 32.8 (10.41) 18, 64 | 32.4 (11.38) 18, 64 | 31.8 (10.15) 18, 64 | 32.1 (10.88) 18, 64 |
| Gender, n (%) | Male Female | 108 (52.7)  97 (47.3) | 69 (49.3) 71 (50.7) | 158 (52.5)  143 (47.5) | 88 (50.6) 86 (49.4) |
| Race, n (%) | Asian Black White Other | 4 (2.0) 170 (82.9) 20 (9.8) 11 (5.4) | 0 (0.0) 124 (88.6) 11 (7.9) 5 (3.6) | 4 (1.3) 260 (86.4) 24 (8.0) 13 (4.3) | 0 (0.0) 155 (89.1) 12 (6.9) 7 (4.0) |
| Ethnicity, n (%) | Hispanic  Non-Hispanic/  Latino  Other | 0 (0.0)  204 (99.5)   1 (0.5) | 0 (0.0)  140 (100.0)   0 (0.0) | 0 (0.0)  299 (99.3)  2 (0.7) | 1 (0.6)  173 (99.4)  0 (0.0) |

**Supplementary Figure 1**

**
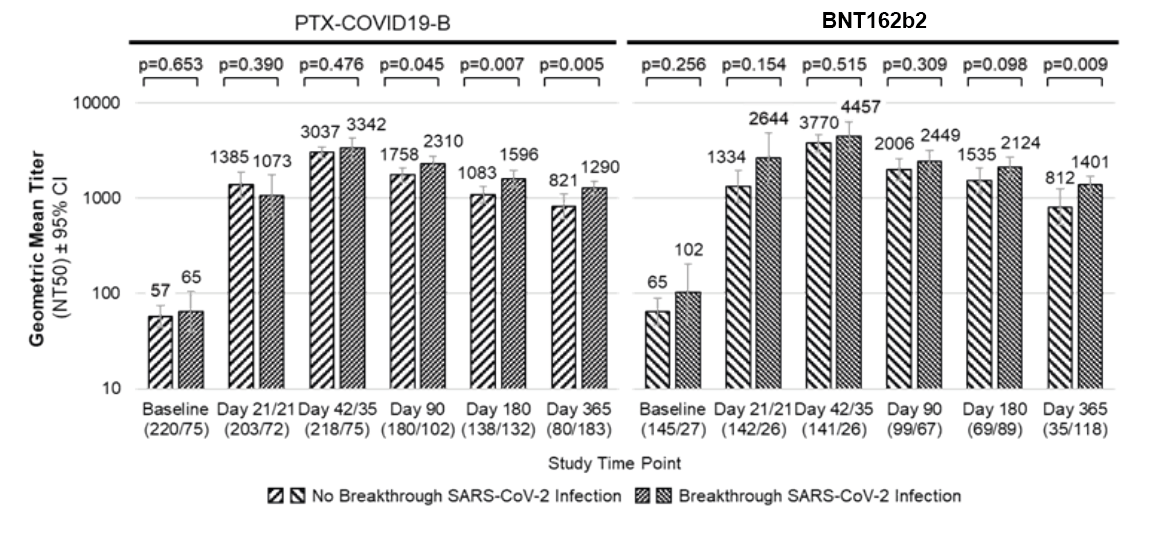
**

**Supplementary Figure 2**


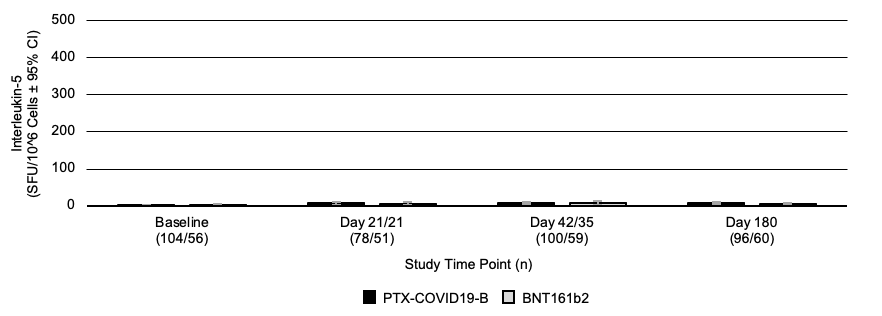

Supplement: Supplementary file 1 — Supplementary Information. [file 41598_2024_55320_MOESM1_ESM.docx]
